# Asking Different Questions: Development and implementation of Clinical and Translational Science Award diversity, equity, and inclusion and community engagement training course for TL1 scholars

**DOI:** 10.1017/cts.2025.36

**Published:** 2025-02-26

**Authors:** Carolina Novella, Desiree M. Sigala, Rachel L. Reeves, Daniel J. Moglen, Frederick J. Meyers, Sarah Rebolloso McCullough, Valentina Medici

**Affiliations:** 1 Feminist Research Institute, University of California Davis, Davis, CA, USA; 2 Clinical and Translational Science Center (CTSC), University of California Davis, Sacramento, CA, USA; 3 UC Davis Comprehensive Cancer Center, University of California Davis, Sacramento, CA, USA; 4 Department of Internal Medicine, Division of Gastroenterology and Hepatology, University of California Davis, Sacramento, CA, USA

**Keywords:** Community engagement, diversity, equity, inclusion, Clinical and Translational Science Award, health disparities, curriculum, social determinants of health, Clinical and Translational Research Training, TL1

## Abstract

**Introduction:**

In recent years, there has been a growth in awareness of the importance of equity and community engagement in clinical and translational research. One key limitation of most training programs is that they focus on change at the individual level. While this is important, such an approach is not sufficient to address systemic inequities built into the norms of clinical and translational research. Therefore, it is necessary to provide training that addresses changing scientific norms and culture to ensure inclusivity and health equity in translational research.

**Method:**

We developed, implemented, and assessed a training course that addressed how research norms are based on histories and legacies of white supremacy, colonialism, and patriarchy, ultimately leading to unintentional exclusionary and biased practices in research. Additionally, the course provides resources for trainees to build skills in how to redress this issue and improve the quality and impact of clinical and translational research. In 2022 and 2023, the course was offered to cohorts of pre and postdoctoral scholars in clinical and translational research at a premier health research Institution.

**Results:**

The efficacy and immediate impact of three training modules, based on community engagement, racial diversity in clinical trials, and cancer clusters, were evaluated with data from both participant feedback and assessment from the authors. TL1 scholars indicated increased new knowledge in the field and described potential future actions to integrate community voices in their own research program.

**Conclusions:**

Results indicate that trainings offered new perspectives and knowledge to the scholars.

## Introduction

In 2021, the National Institutes of Health announced their commitment to end structural racism and racial inequities by supporting diversity, equity, and inclusion (DEI) in funded research and in the biomedical workforce [1]. With this commitment, comes the need to develop and implement educational techniques to provide an understanding of DEI and best practices to address it. There has been a significant interest in DEI training in the medical workforce, with a particular focus on healthcare providers, students, and researchers. Evidence has shown that training has increased cultural sensitivity, awareness of health disparities and the health effects of social, political, and economic structures and has positively impacted the delivery of healthcare and the relationship between providers and patients [2–5]. A study addressing the impact of structural racism training in biomedical research found that the curriculum, consisting of workshops, journal clubs, and movie reviews, was successful at raising institutional awareness of racial and ethnic biases in NCATS-funded TL1 pre and postdoctoral trainees [6]. Additionally, Clinical and Translational Science Award-sponsored KL2 scholars engaged in a health disparities research curriculum designed to increase knowledge and awareness, foster interest, provide skills to evaluate, design and implement disparities research, and foster collaboration in health disparities research [7]. Results indicated that the training was well-received and increased perceived knowledge and competence of health disparities.

DEI trainings such as implicit bias training, focus on changing individual prejudices and stereotypes that lead to discriminatory behavior that impacts patient care and workplace dynamics [8]. These programs tend to focus on increasing awareness of bias at individual and interpersonal levels. There is also a need for training that addresses systemic biases and cultural norms such as the historical, political, and social structures that produce inequities [9]. Examples of these inequitable structures include intergenerational wealth gaps, health care access, disproportionate pollution in communities of color, and histories of exclusion in health science. Failure to address these structural challenges makes it more challenging to address the roots of bias [9]. This training gives participants the opportunity to situate their lived experiences of prejudice and stereotyping within legacies of discrimination that continue up to the present day. This can remove the guilt and defensiveness that sometimes leads to discomfort and prevents participants from fully engaging in training workshops. Instead, participants can understand that though they are not responsible for systems such as racism, they do have a responsibility to help undo their ongoing impacts.

The University of California Davis Clinical and Translational Science Center (CTSC) TL1 clinical and translational training program has been enrolling biomedical research trainees in medical school, graduate students, and postdoctoral scholars in biomedical research since 2006. Given the broad range of trainee levels of education and research interests within clinical and translational research general focus, it was selected as the ideal environment to apply the developed training. In a collaboration between the UC Davis CTSC TL1 program and the Feminist Research Institute (FRI), we hypothesized that a DEI-specific curriculum could target scholars in clinical and translational research and support them in recognizing the impact of historical systems of oppression in research and identifying ways to address this systemic bias. This hypothesis was tested through the development of three training modules which were assessed by post-training surveys. The aims of the present study were to first select training themes rooted in community needs, and second to implement and assess the new training modules among TL1 pre- and post-doctoral scholars.

## Methods

**Figure 1.**
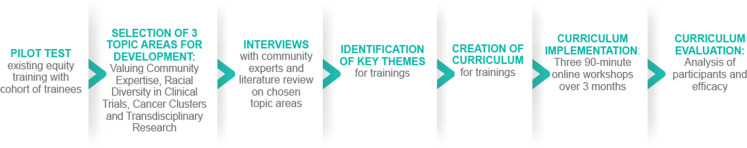
**Methods flowchart.** Methods flowchart from pilot test to the curriculum implementation for TL1 scholars after interviews with community representatives.

Method section outlines the methodology we undertook to create and evaluate the pilot trainings. Results will discuss the implementation and evaluation of the training provided to CTSC TL1 scholars. Fig. 1 represents the workflow of the present study.

### Phase 1: pilot testing of existing training

We pilot-tested three existing training modules from a research training program called Asking Different Questions (ADQ). ADQ is a curriculum designed to teach researchers about how histories of oppression such as white supremacy, colonialism, and patriarchy continue to impact research culture and practices today [10].

Three existing trainings were chosen for the pilot: Making More Accurate Knowledge, Studying Race, Sex & Gender, and Addressing Privilege & Anti-Blackness in Science (see description of each in Supplemental Material 1) (Table 1). These trainings were initially offered remotely via a videoconferencing platform in 2020. At each training, participants listened to a half-hour lecture. They then engaged in small group discussion for an additional half hour, led by a trained facilitator. This format allowed students to have common ground for discussion based on the lecture, while also bringing in their own expertise, both from their professional training and their lived experience. One of the tenets of the training program is that people bring different perspectives and lived experiences to their research, and the diversity of these experiences is valuable. This is borne out of research that has demonstrated the value of having diverse teams [11]. The content of each session described above is drawn from literature in the field of science and technology studies (STS), particularly feminist STS (fSTS) [12–14]. The first session, Making More Accurate Knowledge, reviewed how bias remains in traditional approaches to objectivity, and provided alternative frameworks grounded in fSTS. The second session, Studying Race, Sex, and Gender, provides guidance on how to study these as social constructs within a biomedical setting. The third session, Addressing Anti-Blackness in Science, explores the experience of Black scientists with bias, racism, and stereotyping. Within a week of each session, a closing survey was sent out to all participants via Google Forms. The survey asked if they identified as underrepresented in their field, if they would recommend the session to a colleague, and if they felt the session was relevant to their research field (Table 2). They were also asked two open-ended questions: to elaborate on the impact of the training and provide any additional feedback.

**Table 1.** Initial training modules offered through the pilot study

Training	Number of participants*	Responses in closing survey	Average ratio of learning objectives achieved
Making More Accurate Knowledge	11 for lecture, 4 for discussion	3	2/3
Studying Race, Sex, and Gender	10	4	2/3
Addressing Anti-Blackness in Science	7	5	2/3

**Table 2.** Evaluation of the initial training modules

Question	Number of respondents*	Response
Do you identify as under-represented in your field?	12	Yes: 6 No: 3 Unsure: 3
Would you recommend this training to a colleague?	12	Yes: 12 No: 0
Do you feel that this session was relevant to your research or field?	12	Yes: 12 No: 0

Note: * Some participants attended more than one training module, therefore the number of responses (Table 1) is greater than the number of participants (Table 2).

Six out of the twelve respondents chose to give additional open-ended feedback. All feedback was positive. Three respondents described the sessions as “thought-provoking.” Three respondents said that they want more training like this and to see this training made more widely available to their fellow students.

While those students who gave feedback found these sessions to be highly effective, over half of the attendees did not complete the evaluation. Project leadership also observed significant attrition in the first session between the lecture and the discussion section of the workshop. We posit that discussion may not be a familiar and comfortable pedagogical form for attendees and that the material might have been too advanced for some students. Another challenge is that most attendees had little control over their research agendas, as the agendas were dictated by Principal Investigators who may not have received this sort of training. This limited their ability to enact potential changes suggested in the curriculum. Finally, the curriculum was created for a general STEM audience, not clinical and translational scientists. We hypothesized that a tailored curriculum would be more impactful.

### Phase 2: development of training

The UC Davis CTSC and FRI partnered to create a customized training series addressing histories of exclusion and inequitable practices in health sciences research with the goal to understand how these histories continued to impact contemporary norms and practices. Research from the fields of history of medicine, ethnic studies, science and technology studies, community development, and related fields provided the foundation for the curriculum [15–17]. To select the topics and material covered in the training modules, representative researchers and education specialists from both FRI and CTSC met regularly to determine what would be most applicable and actionable for trainees. The UC Davis Cancer Center planned to have some of their trainees participate. Based on these discussions, the following three topics were chosen for training modules:

1. Valuing Community Expertise: The goal of this module was to teach trainees how histories of medical research systematically devalued the lives, well-being, and expertise of Black communities and communities of color.
2. Racial Diversity in Clinical Trials: The goal of this module was to examine the challenge of racial diversity in clinical trials.
3. Cancer Clusters and Transdisciplinary Research: The goal of this module was to understand the need for transdisciplinary collaboration to address complex sociocultural health issues such as “cancer clusters.”

Researchers often encounter difficulty enrolling diverse populations in clinical trials. We conducted a literature review [16,18–20] and interviewed four expert community leaders. Using purposive sampling, we chose two clinical faculty, one community researcher, and one *promotora de salud* (community health worker). These interviews were conducted in 2022 both via videoconference and in person. Interview questions included, “what is your experience with clinical trials recruitment?” “Which are some factors that you believe hinder or foster diversity and recruitment among communities?.” Key themes from the interview and literature reviews were the importance of attending to histories of harm, avoiding applying deficit-based frameworks to communities, and showing humility when working with target communities.

Cancer clusters are examples of systemic health inequities that can be challenging to study without taking sociopolitical systems into account. Our project team interviewed two members of the UC Davis Environmental Justice Fellows Program. These fellows, individuals working at the crossroad of community health and environmental advocacy, provided a critical lens from their experience working in the field. In the open-ended interviews with these individuals, we asked what they would stress in a training for scientists on community health and environmental justice. Key themes were listening to and believing community stories, addressing root causes, and working for systemic change.

The post-training evaluations were developed by FRI. Learning objective questions were designed to measure three levels of learning based on Bloom’s Taxonomy: comprehension, analysis, and application [21]. FRI leadership chose to ask about relevancy and recommendation to peers to understand how applicable the training felt for them, and thus its potential efficacy. We ask if participants are underrepresented rather than demographic data (gender/race) because what constitutes underrepresented can shift from field to field.

## Results

A total of 48 TL1 scholars participated in the trainings (n = 19 to module 1; *n* = 16 to module 2; *n* = 13 to module 3).

### Phase 3: curriculum implementation

Each module was 1.5 hours in length. Modules had learning objectives that assessed comprehension and application, both intellectually and in practice. Based on Bloom’s Taxonomy, these learning objectives assessed understanding of the topic discussed, ability to apply the knowledge to their research context, and ability to take action or change practices based on what they learned [21]. The modules were created to first provide trainees with an understanding of new concepts and knowledge that would deepen their understanding of the role of inequity in health sciences. This was done through half-hour lecture presentations. Modules include also case studies to better understand the mechanisms by which a research agenda can focus on the individual instead of a system. Then, they sought to provide trainees with the tools to apply this new understanding to their own research and field and to identify specific actions they could take to address the inequities discussed. This was achieved through 30–45 minute facilitated small group discussions. These discussions were led by trained peer facilitators.

### Phase 4: curriculum evaluation and feedbacks

Participants were required by their program to take part in at least one session. Real-time polls, feedback surveys by participants, and follow-up questionnaires provided evaluation data. The modules featured real-time online polls for participants used to engage the participants as well as to guide later evaluation efforts. Additionally, participants responded to feedback surveys related to their experiences of attending the training, right after the training.

Aferward, lectures were edited into videos posted on video sharing website (YouTube). A resource guide was distributed among participants. This resource guide contained a bibliography organized by topics discussed in each training and a link to a YouTube video of the lecture portion of the training. This offered participants the opportunity to follow up with provided material as needed in their future careers.

An evaluation of learning objectives was distributed to all participants (see Supplementary Material 2 for list of questions and multiple-choice responses). The response rate was close to 100% and reported in detail in Table 3.

**Table 3.** Response rate for Asking Different Questions (ADQ) module evaluations

	N evaluation respondents/N module participants (rate %)	
	2022	2023
**Module 1**	9/9 (100%)	10/11 (90%)
**Module 2**	6/6 (100%)	10/10 (100%)
**Module 3**	3/3 (100%)	10/10 (100%)

The evaluation showed that nearly all participants achieved learning goals that involved comprehension of new concepts. This is learning that involves understanding new concepts. Examples include “I understand the difference between framings of individual deficits vs. systemic deficits in diversifying clinical trials” and “I can identify ways that their research can engage communities.” The majority (81%) achieved the next level of learning – application – as indicated by their affirmative responses to “I can apply the concept of two-way trust to identify areas of growth for myself and my research.”

**Figure 2.**
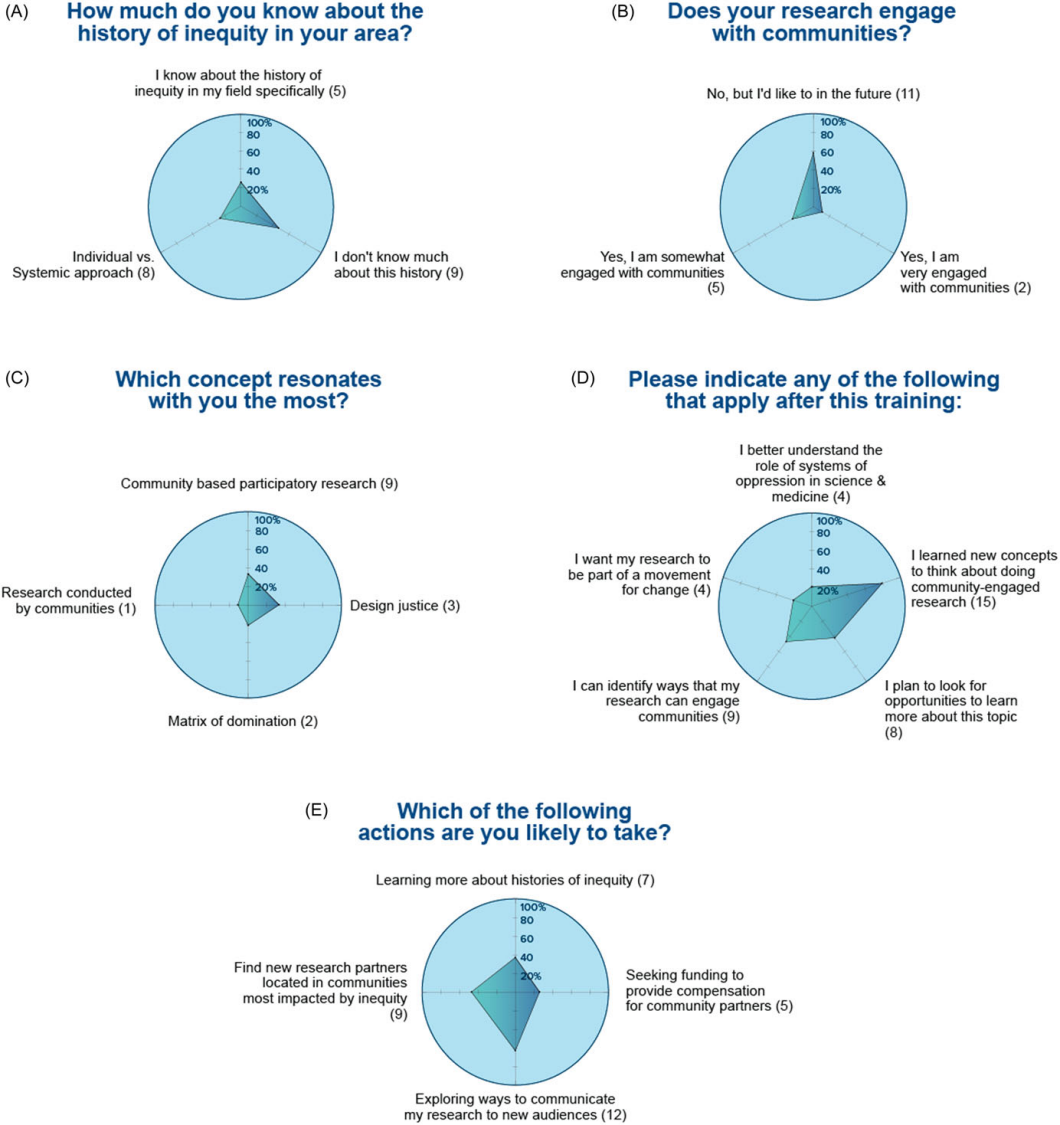
**Module 1. Valuing community expertise.** Nineteen trainees participated to this module and responded to the following questions: (A) how much do you know about the history of inequity in your area?; (B) does your research engage with communities?; (C) which concept resonates with you the most?; (D) indicate which applies after the training; and (E) which of the following actions are you likely to take?

About half of the scholars who attended Module 1 on “Valuing community expertise” reported that their research is not engaged with or is only somewhat engaged with any community (Fig. 2A) and they acknowledged poor knowledge about history of inequities in their research field (Figure 2B). However, they also claimed the willingness to take action to find new research partners within communities most impacted by inequity and to identify funding to compensate community partners (Figure 2C).

**Figure 3.**
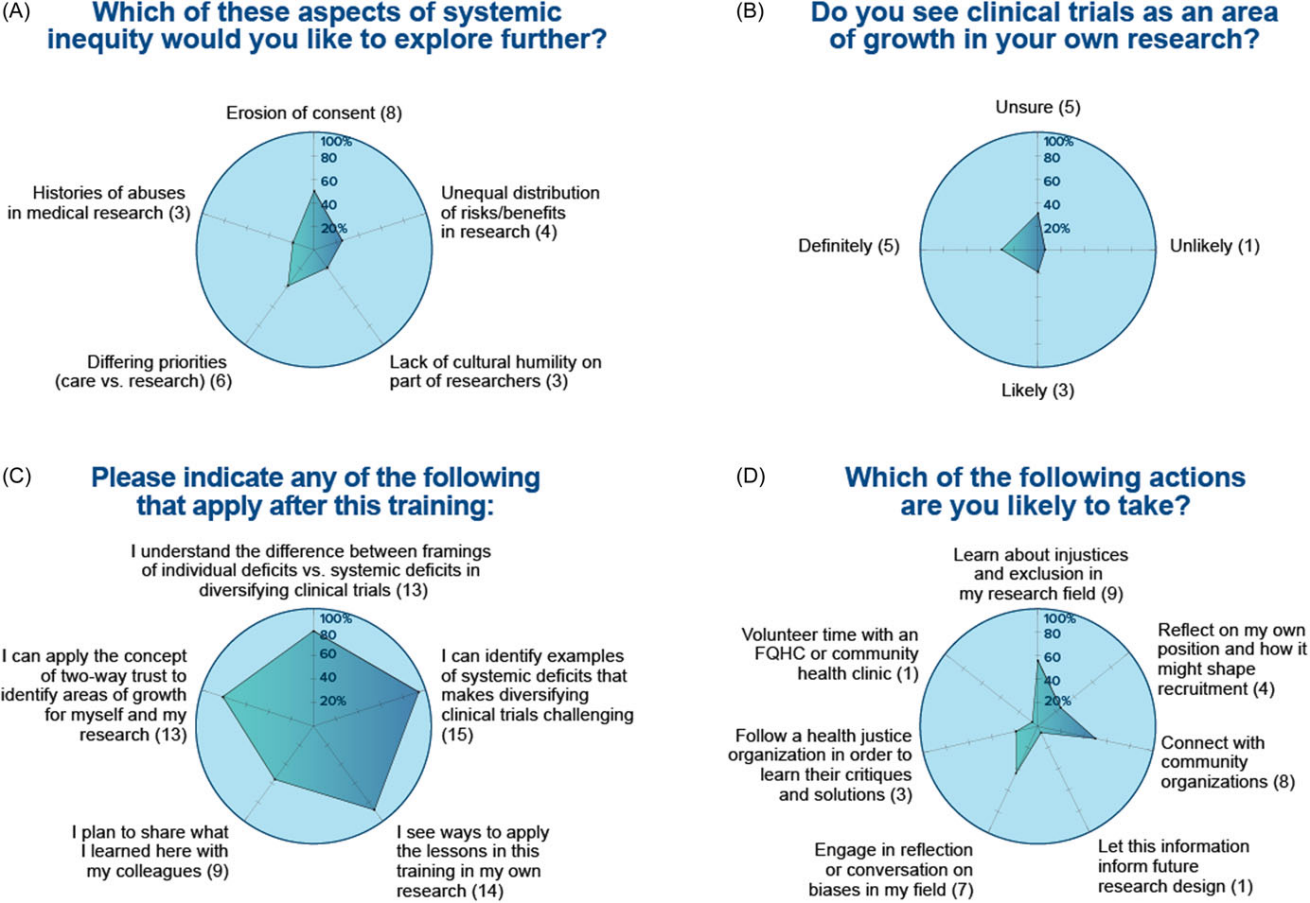
**Module 2. Racial diversity in clinical trials.** Sixteen trainees participated to this module and responded to the following questions: (A) how much do you know about the history of inequity in your area?; (B) do you see clinical trials as an area of growth in your own research?; (C) indicate which applies after the training; and (D) which of the following actions are you likely to take?

**Figure 4.**
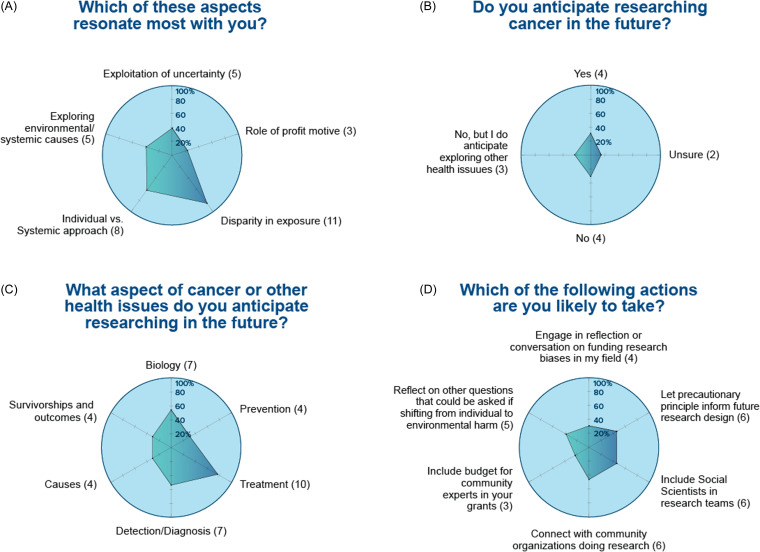
**Module 3. Cancer clusters, environmental justice and transdisciplinary research.** Thirteen trainees participated to this module and responded to the following questions: (A) which of these aspects resonate most with you?; (B) do you anticipate researching cancer in the future?; (C) what aspect of cancer or other health issues do you anticipate researching in the future?; and (D) which of the following actions are you likely to take?

Similarly, the survey regarding Module 2 on Clinical trials (Fig. 3) showed about half of the scholars will expand their research to include clinical trials and plans and have learned information that they will apply to their own research. Module 3 on Cancer Clusters (Figure 4) showed that, despite only 25% of students were currently conducting cancer research, most of them will take tangible actions including adding social scientists in their research team, planning for budget in their research grants to support community experts, or making connections with community organizations doing research.

## Discussion

The main findings of our study include the successful development and positive evaluation of DEI-specific training modules. Importantly, the pilot-tested training was developed using a community engagement approach. The three training modules were implemented among pre- and post-doctoral scholars and the evaluation showed a positive assessment with potential to change long-term approaches to research, confirming our starting hypothesis that a DEI-specific curriculum could be designed targeting trainees in clinical and translational research and support them in the development of their projects.

The aim of this study was to develop trainings rooted in community needs. The designed trainings centered on challenging systemic inequity in clinical and translational research, ultimately reorienting the “problem.” When the problem is imagined, implicitly or explicitly, as residing in the under-represented community, then the answer is often one that requires them to change their attitude, thoughts, beliefs, and actions. Solutions tend to point towards education or persuasion. When the problem is framed as residing in the medical system, then we look to change aspects of the system as the solution. Thus, we made this differentiation in framing explicit, demonstrating how an individual/community problem approach versus a systemic approach rests on different assumptions, leading to different solutions and actions on the part of recruitment teams and researchers. Some researchers have referred to this as moving from a model of individual/community deficit to a model of systemic deficit or inequity [22,23].

Participants also learned that examining racial diversity in clinical trials is important because it serves as a way to account for the impacts of trauma and everyday stressors that result from systemic racism, both within the health care system [24,25] and society at large. Race is not a biological phenomenon subject, but rather a complex social construct with physiological patterns and impacts [26].

One example of this shift can be seen in how the training taught the “complexity of trust.” Rather than just looking at ways to gain trust from racialized communities, the training suggested that trainees consider “What have we done to demonstrate that the same institutions that perpetuated historical abuses are now trustworthy?.” Building trust requires the acknowledgment and accountability for past damages, clear signs of addressing current inequities, and mechanisms for considering the priorities of the communities in research. One *promotora* interviewed said, “Las comunidades [11] would trust if they were approached in the right way.” The “right way” requires genuine connection, listening, and trusting that the community knows what it needs.

The training introduced a model of “moving beyond cultural competency” based on conversations with the interviewees and research conducted. Cultural competency approaches tend to focus on how a culture can be accessed, learned, and organized in a training, or addressed by hiring one representative of the specific group. This approach can be an impediment because it can lead to a “fixed” understanding of the community. Rather than focusing on researcher mastery of cultural knowledge, trainees were taught to learn “cultural humility,” which involves respect, genuineness, and an openness to learning from the community [20,27].

Trainees learned how this approach stands in contrast to “parachute” researchers who drop into a community, gather the data they need, and disappear. These methods do not speak to community needs. Instead, community members want to work with researchers to generate data and analysis that will serve immediate community needs and goals. Combining the expertise of community leaders with research expertise, particularly from multiple fields, can produce powerful results.

These community-engaged methods that respond to community needs are more successful in improving participation in clinical trials [28,29]. For example, when community health workers administered health needs assessments, provided medical and social referrals, and gave participants links to relevant research studies, they saw an increase in clinical trial participation, particularly among Black men [30].

Cancer Clusters, Environmental Justice, and Transdisciplinary Research unit is focused on the challenge of health inequities and their relationship to environmental inequities and sociocultural issues. Trainees were taught that “cancer clusters” is a term used often by community activists to describe their experience of higher cancer rates in a particular region or around a specific environment. These increased rates are often attributed to pollution affecting their local environment and derived from industry or military sites. This is a specific example of a larger phenomenon of health inequities, particularly in communities of color. Health issues including asthma, cardiovascular diseases, and diabetes may occur in these communities in association with the higher levels of pollution [31–34]. Trainees learned how communities often mobilize to prevent these harms by advocating for changes to the practices of those polluting their environment [35–37]. However, clearly linking these harms to specific industries or pollution sites can be challenging. The lack of certainty in research, which is an essential part of the scientific process, can be exploited by parties wishing to sow doubt [38]. In other words, industries that may be releasing pollutants often fight against any changes that may harm their operations or financial outlook. This ultimately leads to the exploitation of uncertainty [39]. Adhering to the precautionary principle rather than utilizing threshold assessment can help mitigate this challenge [40–42].

Community experts working in communities with health inequities advocated for systemic changes rather than a focus on individual choice. Much of cancer and disease prevention relies upon individual risk management. This model presumes a level of individual autonomy which many in frontline communities do not have as they are subjected to environmental injustices such as poor air pollution, sub-par water quality, and circulation of toxins. Many do not want to move, as they do not wish to abandon their community. They may not have viable access to quality health care due to lack of transportation, economic burdens, or an overwhelmed local healthcare system. They may also have limited access to healthy food options, safe places to exercise, and green space to recreate – keys to improving individual health. Thus, the “choice” to engage in behavior change such as healthier eating or exercising is complicated by environmental factors outside their control.

The training used the example of breast cancer to offer a partial explanation for this imbalance. Breast cancer funding dedicated to prevention via environmental interventions is relatively low [39,43]. Individual interventions, which see much larger investment, offer a lucrative market to grow profit margins in the form of prevention campaigns, new testing methods, and treatment technologies. In contrast, environmental interventions can threaten profit margins, as they could result in more stringent regulations for the release of chemical byproducts and toxins into the environment and in products.

This training showed that participants had significant interest in building relationships with impacted communities and having the needs of those communities influence their research. In the module on Clinical Trials, 50% of attendees planned to connect with community organizations, and the majority were interested in working with communities. This is notable and likely reflects a commitment to doing research that improves the needs of vulnerable populations. This can improve the quality and rigor of research [44]. And yet, it was evident that trainees had very little training on how to do this well, indicated by how little they said they knew about history of inequity in their field. Best practices in community engagement show how important knowledge of this history is [45]. Though an in-depth history of inequity was not the focus of the training, 79% of participants learned new concepts to think about doing community-engaged research. In the module on Racial Diversity in Clinical Trials, 56% of attendees planned to learn more about injustices and exclusions in their field. However, they did not express the same level of interest in learning more, with only 50% planning to learn more about community engagement and 39% planning to learn more about histories of inequity. This indicates a disjunction between desire to perform community-engaged research and desire to learn information that would better equip them to do this work.

Further research is needed to assess the impact of training on community involvement in clinical and translational research. This training was designed to provide more than just a how-to; it also sought to teach participants the roots of inequities that led to the exclusion of community voices in medicine. Teaching the origins both gives trainees the knowledge to understand the depth of the challenge and a foundation from which to engage in critical thinking to find new, creative solutions. Given the small sample size and short duration of the training, we cannot assess generalizable results nor mid- to long-term outcomes. Development in study design could also yield more feedback.

Our approach presents a few limitations. We conducted only post-intervention surveys but Improved evaluation methods should include pre- and post-intervention attitudinal and motivational surveys, follow up surveys to assess actions taken, or discussion analysis to assess achievement of learning outcomes. This evaluation should be designed beforehand, to establish a collaborative decision-making process to define goals, desired changes, and measurable actions. These measurable actions need to consider not only individual perceptions but also institutional change. Experimental designs that include control groups would offer grounds for further developing these kinds of training and potential indicators could be the number of relations developed with the community and follow-up consultations. In addition, different types of scholars and different levels of training should be included. We included medical students, graduate students, and post-doctoral fellows, but the training should be extended to faculty and research administrators. There is also need for long-term follow up and outcome assessment to evaluate how scholars implemented the training in their research practices. An additional limitation is that our study was conducted in a single center, academic institution.

The greatest impact of this study is the development of a new model to conduct transdisciplinary collaboration between social sciences/humanities and medical sciences. This innovative training sought to apply well-established insights from the study of biomedicine and research to the specific context of clinical and translational medicine training, while also creating a mechanism for the training to be informed by local community health professionals.

Along with further research indicated in the discussion, there is also a benefit to exploring the creation of further training on issues including studying race and gender in biomedical research, as sociocultural constructs with biophysical impacts.

As the UC Davis CTSC TL1 program continues to provide the described training, the modules will be offered to all trainees, including research faculty and research staff within the CTSC, and further instruments for pre- and post-training assessment will be implemented.

## Supporting information

Novella et al. supplementary material 1 Novella et al. supplementary material

Novella et al. supplementary material 2 Novella et al. supplementary material

## Supplementary material

The supplementary material for this article can be found at https://doi.org/10.1017/cts.2025.36.
