# Supplementary material for: Asking Different Questions: Development and implementation of Clinical and Translational Science Award diversity, equity, and inclusion and community engagement training course for TL1 scholars
Source: J Clin Transl Sci. 2025 Feb 26;9(1):e69. doi: 10.1017/cts.2025.36 (PMC11975778; doi:10.1017/cts.2025.36)
Supplement: Novella et al. supplementary material 1 — Novella et al. supplementary material [file S2059866125000366sup001.docx]

**Supplemental Material**

Description of Trainings Selected for Pilot

1. **Making More Accurate Knowledge**: How can more nuanced models of objectivity allow us to more accurately represent research findings? Scientific findings are often confused and situated in specific historical, institutional, and cultural conditions that are not acknowledged in published findings. Participants will be introduced to how models of objectivity have changed over time within the scientific community and learn about potential new, more accurate models that account for how science and culture interact.
2. **Studying Race, Sex, and Gender:** How can we study race, sex, and gender in ways that are more precise to produce better research results? Race, gender, and even sex are sociocultural constructs. And yet, they have real impacts on our daily lives and well-being. How can researchers best take these important identity markers into account without succumbing to a false biological determinism? This module will identify common pitfalls that undermine research findings and identify more productive pathways for studying race, sex, and gender in research.
3. **Addressing Privilege & Anti-Blackness in Science:** What are we doing to address ongoing anti-Blackness and amend for generations of exclusion? How must our departments, professional organizations, research systems, mentoring practices, and administrative structures change? We will share resources to learn and identify sites for personal and collective action. This module will augment our efforts toward self-education and create community in our efforts to redress the legacies of white supremacy in academia.
