# Supplementary material for: Asking Different Questions: Development and implementation of Clinical and Translational Science Award diversity, equity, and inclusion and community engagement training course for TL1 scholars
Source: J Clin Transl Sci. 2025 Feb 26;9(1):e69. doi: 10.1017/cts.2025.36 (PMC11975778; doi:10.1017/cts.2025.36)
Supplement: Novella et al. supplementary material 2 — Novella et al. supplementary material [file S2059866125000366sup002.docx]

**Supplemental Material 2.**

| **Module 1. Valuing Community Expertise** | | |
| --- | --- | --- |
| **Questions** | **Responses** | **Rationale** |
| **How much do you know about the history of inequity in your area?** | 1. I don't know much about this history 2. I know about the history of inequity in my field specifically 3. Individual vs. systemic approach | Encourages reflection on current knowledge. Allows participants and instructor to assess pre-existing understandings. |
| **Does your research engage with communities**? | 1. No, but I'd like to in the future 2. Yes, I am very engaged with communities 3. Yes, I am somewhat engaged with communities | Encourages reflection on current community engagement practices. Allows participants and instructor to assess pre-existing practices. |
| **Which concept resonates with you the most?** | 1. Community bases participatory research 2. Research conducted by communities 3. Design justice 4. Matrix of domination | Encourages reflection on concepts presented in training. Provides instructor feedback on most impactful concepts. |
| **Please indicate any of the following that apply after this training:** | 1. I better understand the role of systems of oppression in science & medicine 2. I learned new concepts to think about doing community-engaged research 3. I plan to look for opportunities to learn more about this topic 4. I can identify ways that my research can engage communities 5. I want my research to be part of a movement for change | Assesses learning outcomes at different levels in Bloom’s taxonomy. Evaluates motivation to enact lessons of training. |
| **Which of the following actions are you likely to take?** | 1. Learning more about histories of inequity 2. Seeking funding to provide compensation for community partners 3. Exploring ways to communicate my research to new audiences 4. Find new research partners located in communities most impacted by inequity | Assesses impact of goal to incite action in participants. Determines areas of greatest impact on participants. |

| **Module 2. Racial Diversity in Clinical Trials** | | |
| --- | --- | --- |
| **Questions** | **Responses** | **Rationale** |
| **Which of these aspects of systemic inequity would you like to explore further?** | 1. Erosion of consent 2. History of abuses in medical research 3. Differing priorities (care vs research) 4. Lack of cultural humility on part of researchers 5. Unequal distribution of risks/benefits in research (phase I-phase III trials) | Determines areas of interest of participants. |
| **Do you see clinical trials as an area of growth in your own research?** | 1. Unsure 2. Unlikely 3. Likely 4. Definitely | Determines applicability of training focus to audience. |
| **Please indicate any of the following that apply after this training:** | 1. I understand the difference between framings of individual deficits vs. systemic deficits in diversifying clinical trials 2. I can apply the concept of two-way trust to identify areas of growth for myself and my research. 3. I plan to share what I learned here with my colleagues 4. I can identify examples of systemic deficits that makes diversifying clinical trials challenging 5. I see ways to apply the lessons in this training in my own research | Assesses learning outcomes at different levels in Bloom’s taxonomy. Evaluates motivation to enact lessons of training. |
| **Which of the following actions are you likely to take?** | 1. Learn about injustices and exclusion in my research field 2. Volunteer time with an FQHC or community health clinic 3. Follow a health justice organization in order to learn their critiques and solutions 4. Engage in reflection or conversation on biases in my field 5. Let this information inform future research design 6. Connect with community organizations 7. Reflect on my own position and how it might shape recruitment | Assesses impact of goal to incite action in participants. Determines areas of greatest impact on participants. |

| **Module 3. Cancer clusters, environmental justice and transdisciplinary research** | | |
| --- | --- | --- |
| **Questions** | **Responses** | **Rationale** |
| **Which of these aspects resonate most with you?** | 1. Exploitation of uncertainty 2. Exploring environmental/systemic causes 3. Individual vs. systemic approach 4. Disparity in exposure 5. Role of profit motive | Encourages reflection on concepts presented in training. Provides instructor feedback on most impactful concepts. |
| **Do you anticipate researching cancer in the future?** | 1. Yes 2. Unsure 3. No 4. No, but I do anticipate exploring other health issues | Determines applicability of training focus to audience. |
| **What aspect of cancer or other health issues do you anticipate researching in the future?** | 1. Biology 2. Survivorships and outcomes 3. Causes 4. Prevention 5. Detection/Diagnosis 6. Treatment | Determines applicability of training focus to audience. |
| **Which of the following actions are you likely to take?** | 1. Engage in reflection or conversation on funding research biases in my field 2. Let precautionary principle inform future research design 3. Include Social Scientists in research teams 4. Connect with community organizations doing research 5. Include budget for community experts in your grants 6. Reflect on other questions that could be asked if shifting from individual to environmental harm | Assesses impact of goal to incite action in participants. Determines areas of greatest impact on participants |
